# Evaluation of the GenoType Mycobacterium CM/AS Assay for Species Identification of Mycobacteria

**DOI:** 10.7759/cureus.90958

**Published:** 2025-08-25

**Authors:** Mandira Ramudamu, Bharti Malhotra

**Affiliations:** 1 Microbiology, Madha Medical College and Hospital Kovur, Chennai, IND; 2 Microbiology, Sawai Man Singh Medical College Jaipur, Jaipur, IND

**Keywords:** dna strip assay, genotype mycobacterium cm/as assay, mtb (mycobacterium tuberculosis), nontuberculous mycobacteria (ntm), reverse hybridization

## Abstract

Background: The early differentiation of *Mycobacterium tuberculosis *(MTB) from nontuberculous mycobacteria (NTM) and the identification of species among NTM are crucial for the immediate implementation of the appropriate therapy because susceptibility to drugs varies widely among different species. Identification to the species level by classical biochemical methods is time-consuming, requires a large battery of tests to be run, and results are obtained in four to six weeks of obtaining the isolate. The introduction of molecular biological methods has greatly improved the speed and accuracy of the process. Recently, DNA strip assays for the identification of *Mycobacterium* to the species level have been developed. These assays are based on reverse hybridization of a polymerase chain reaction (PCR) product to a nitrocellulose strip with immobilized probes for different mycobacterial species. One such assay is GenoType Mycobacterium CM/AS assay (Hain Lifescience GmbH, Nehren, Germany).

Aim: To evaluate the diagnostic accuracy of the GenoType Mycobacterium CM/AS assay for the species identification of mycobacteria in the culture isolates in comparison with conventional phenotypic and biochemical methods.

Material and methods: A total of 160 mycobacterial isolates on solid Lowenstein-Jensen media or liquid MGIT 960 were subjected to species identification by GenoType Mycobacterium CM/AS assay and biochemical methods.

Results: Sensitivity, specificity, positive predictive value (PPV) and negative predictive Value (NPV) were found to be 100% for all the mycobacterialisolates except *M. terrae*, where sensitivity and NPV were 0% but specificity was 100% and PPV was 98.13%. Hence the overall sensitivity of the GenoType Mycobacterium CM/AS assay was 98.13% and the specificity was 100%.

Conclusion: The GenoType assay is a simple, rapid and reliable method for the identification of clinically important mycobacteria, and it is well suited for use in a routine laboratory.

## Introduction

The genus *Mycobacterium *consistsof* *acid fast, aerobic bacilli belonging to the family Mycobacteriaceae and is one of several mycolic acid-containing genera within the order Actinomycetales. The genus comprises around 200 species, broadly categorized into the *M. tuberculosis *complex (MTBC) and nontuberculous mycobacteria (NTM) [1]. 

NTM, also known as “atypical mycobacteria”, nontuberculous, opportunistic or mycobacteria other than tubercle bacilli (MOTT), have been known for a long time but have been overshadowed by MTB and dismissed as contaminants. Clinical significance has now been appreciated as NTM are becoming recognized as true pathogens by many authors [2]. In India, by the year 2000, reports of NTM isolation and disease association had already begun to increase, reflecting their rising clinical relevance. Overall NTM isolation rate varied from 0.7% to 34% from different parts of India [3] and 3.6% to 12.4% from the northwest region of India [4]. 

It is important to identify the species of NTM, as the clinical signs and symptoms are similar to those of MTB but require different antimicrobials as per species, as most of them are highly resistant [5,6].

Failure to diagnose NTM infection and treatment of the patient as having tuberculosis (TB) may have serious consequences. Most NTM are resistant to the commonly used anti-tuberculous drugs and failure to respond to inappropriate treatment with these drugs may lead to the patient being misdiagnosed as having drug-resistant TB and result in further exposure to ineffective agents with serious side effects. The number of patients with NTM infection is increasing and moreover there is strong geographical variation in the responsible NTM species [7].

So, early and reliable identification of mycobacteria will help in initiating proper treatment and may spare patients from unnecessary treatment in the case of growth of environmental contaminants. To confirm that the isolated NTM is responsible for infection, it is important to isolate the same species at least three times. Identification to the species level by classical phenotypic and biochemical methods is time-consuming, requires a large number of battery of tests to be performed and results are obtained in four to six weeks after obtaining the isolate. It has been recognized that the use of liquid medium significantly increases isolation rates and decreases the recovery time of mycobacteria but biochemical reactions require isolates from solid media and therefore necessitate additional subculture of isolates obtained from liquid cultures [8].

Newer molecular methods have greatly improved the speed and accuracy of species identification like DNA sequencing, pyrosequencing, polymerase chain reaction-restriction fragment length polymorphism (PCR-RFLP) assays, real-time PCR assays, oligonucleotide arrays, AccuProbe (Gen-Probe Inc., San Diego, CA, USA), line probe assays (LPA), etc. Most of these methods require either expensive equipment or extensive expert knowledge or are restricted to a limited number of species that can be identified [9].

Recently, DNA strip assays have been developed, GenoType Mycobacterium CM/AS assay and GenoType MTBC assay (Hain Lifescience GmbH, Nehren, Germany). These assays are based on reverse hybridization of a PCR product to a nitrocellulose strip with immobilized probes for different mycobacterial species [8].

The GenoType Mycobacterium CM/AS assay consists of two assays, the common mycobacteria (CM) and the additional species (AS), which are executed consecutively to identify NTM. The CM assay enables the simultaneous identification of species, including the most relevant *M. tuberculosis* complex, members of the *M. avium *complex, *M. kansasii *and *M. chelonae*. Additionally, the AS test provides the identification of species that are found more infrequently, such as *M. simiae, M. mucogenicum* and *M. celatum *[10]. 

The aim of this study was to evaluate the diagnostic accuracy of the GenoType Mycobacterium CM/AS assays for the species identification of *Mycobacterium *in the culture isolates in comparison with conventional phenotypic and biochemical methods.

## Materials and methods

Study design and sample size

This was a descriptive, cross-sectional study conducted in the Advanced Research and Mycobacteriology Laboratory, Department of Microbiology, S.M.S. Medical College, Jaipur, Rajasthan, over an 18-month period. The study included 160 mycobacterial isolates obtained from respiratory samples after approval from the Institutional Research Review Board and Ethics Committee (Approval No: 558 MC/EC). The sample size was calculated prospectively using Buderer’s formula for diagnostic-accuracy studies, assuming sensitivity and specificity of 95%, a 95% confidence level, and a precision of ±5%. This yielded a minimum requirement of 146 isolates; allowing for possible culture loss, the final target was set at 160 isolates.

Methods

Clinical specimens suspected of TB/multidrug-resistant (MDR)-TB were cultured on both Lowenstein-Jensen (LJ) medium and in MGIT 960 liquid culture system as per routine laboratory protocol. Any isolate that was culture-positive on either LJ or MGIT was included in the study. All culture-positive isolates were first subjected to Ziehl-Neelsen staining, and acid-fast bacilli (AFB) positive isolates were subjected to the MPT64 antigen test to differentiate MTB and NTM. Thereafter the isolates were processed for species identification by conventional/biochemical methods and GenoType CM/AS assay. Laboratory personnel were not blinded to the initial MPT64 antigen test; however, interpretation of the GenoType CM/AS assay was template-based and objective, while biochemical tests were performed independently to minimize bias. 

All sample processing was performed in a Biosafety Level 3 (BSL-3) certified laboratory, as required for handling specimens suspected of containing *Mycobacterium tuberculosis*. Biochemical tests were carried out inside a Class II, Type A2 biosafety cabinet, in accordance with recommended biosafety guidelines.

Table 1 shows the series of biochemical tests that were done for speciation of NTM [11].

**Table 1:** Series of biochemical tests done for speciation of nontuberculous mycobacteria

S. No.	Biochemical Test
1	Para Nitro Benzoic Acid (PNB) Test
2	Growth Rate
3	Pigment Production
4	Niacin Production Test
5	Nitrate Reduction Test
6	5% NaCl Tolerance Test
7	Heat-Stable Catalase Test at 68°C
8	Semiquantitative Catalase Test
9	Urease Test
10	Tween 80 Hydrolysis Test

GenoType CM/AS assay

The GenoType Mycobacterium CM/AS assays were carried out according to the manufacturer’s instructions, using the reagents provided with GenoType kits. Protocols consisted of DNA extraction by a kit (Genolyse 1.0; Hain Lifescience) followed by PCR amplification, hybridization of the PCR products to the strips, and detection and interpretation of the results [12].

1 ml of suspension of isolate was centrifuged and processed for DNA extraction by Genolyse. The supernatant (DNA) was then transferred to another labelled Eppendorf tube and 5 µl of this DNA was used for amplification. The total number of samples to be amplified was determined. The master mix (Genolyse 1.0) containing all the reagents was prepared and mixed well. The master mix contained 35 µL of pre-nucleotide mix (PNM), 5 µl of 10x PCR buffer, 2 µl of 25 mM MgCl2, 0.2 µl (1 U) Taq polymerase and 3 µl molecular grade water. 45 µl of master mix was aliquoted into each of the prepared PCR tubes. 5 µl of extracted DNA was added to the master mix and mixed well. This was then subjected to amplification in thermal cycler. 

**Figure 1:**
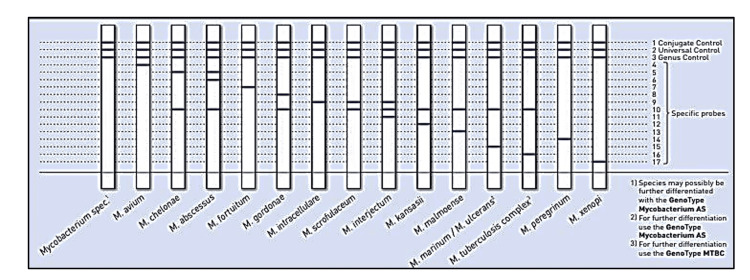
GenoType Mycobacterium CM interpretation sheet In kit insert: bands 7, 14 also represent *M. fortuitum*; bands 5, 10 and 5, 6, 10 also denote *M. immunogenicum*; bands 10, 13 apart from *M. malmoense *represent *M. haemophilum*. Source: Hain Life Science GenoType Mycobacterium CM/AS assay Instruction manual [12].

**Figure 2:**
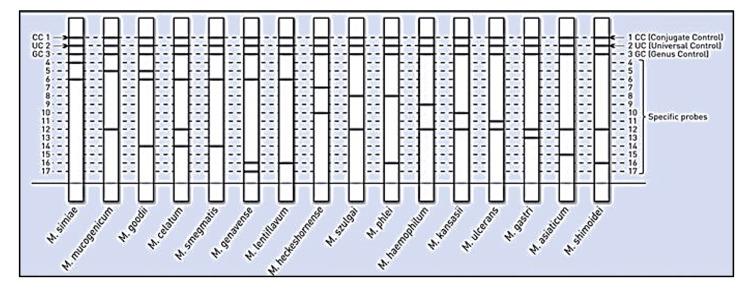
GenoType Mycobacterium AS interpretation sheet Conjugate control – checks the binding of the conjugate on the strip and a correct chromogenic reactions; Universal control – detects all mycobacteria and members of the group of gram positive bacteria with a high G + C content; Genus control – documents the presence of a member of the genus Mycobacterium. Source: Hain Life Science GenoType Mycobacterium CM/AS assay Instruction manual [12].

Amplicons generated were then subjected to hybridisation by GenoType Mycobacterium CM/AS assay. Strips obtained were pasted and results interpreted according to the evaluation sheet as shown in Figures 1, 2.

Quality control

Quality control was ensured at multiple stages. The Genolyse DNA extraction kit includes an internal lysis control to validate the extraction process. In addition we included two extraction controls. The PCR master mix for the GenoType assay contains an internal amplification control to detect PCR inhibition. For the final hybridization step, each strip contains a conjugate control (CC) to verify the reaction, a universal control (UC) to detect mycobacteria, and a genus control (GC) to confirm the Mycobacterium genus. A valid result required the presence of all specified control bands. For biochemical tests, standard American Type Culture Collection control strains and known NTM strains were run in parallel with each batch of clinical isolates.

Statistical analyses

Statistical analyses were performed using SPSS version 20 (IBM Corp., Armonk, NY, USA) and PRIMER (PRIMER-e, Auckland, New Zealand). Categorical data are presented as proportions/percentages. Agreement between biochemical methods and the CM/AS assay was assessed using the concordance rate (overall agreement, %). Diagnostic accuracy measures-sensitivity, specificity, positive and negative predictive values-were calculated for the CM/AS assay against conventional biochemical methods as the comparator, with 95% confidence intervals.

## Results

A total of 160 mycobacterial isolates were subjected to phenotypic/biochemical reactions and GenoType Mycobacterium CM/AS assay for species identification.

The phenotypic and biochemical characteristics of the identified *Mycobacterium *species and the distribution of* *isolates (n=160) are summarized in Tables 2, 3.

**Table 2:** Phenotypic and biochemical characteristics of Mycobacterium species Growth rate test: S – slow growers, R – rapid growers Pigmentation test: P – photochromogen, S – scotochromogen, N – nonphotochromogen Other test results: V – variable, +/- positive/negative

Groups	Species	Growth Rate	Pigmentation	Niacin	Nitrate	5% NaCl	68^o^C Catalase	Semiquantitative Catalase	Urease	Tween 80
M. tuberculosis complex	M. tuberculosis	S	N	+	+	-	-	-	+/-	-
Photochromogens	M.kansasii	S	P	-	+	-	+	+	+	+
	M.simiae	S	P	+/-	-	-	+	+	+	V
Scotochromogens	M.scrofulaceum	S	S	-	-	-	+	+	+	-
Non- Photochromogens	M. avium/ intracellulare	S	N	-	-	-	V	-	-	-
	M. malmoense	S	N	-	-	-	V	-	V	+
	M. terrae	S	N	-	V	-	+	+	-	+
Rapid Growers	M. fortuitum	R	N	-	+	+	+	+	+	V
	M. chelonae	R	N	V	-	-	+	+	+	V
	M. abscessus	R	N	-	-	+	+	+	+	V

**Table 3:** Distribution of Mycobacterium isolates based on phenotypic characteristics and biochemical reactions (n=160)

S. No.	Group		Species	Number	Percentage
1	Slow growers 40 (25%)	Photochromogens 26 (16.25%)	M. kansasii	16	10%
			M. simiae	10	6.25%
2		Scotochromogens 2 (1.25%)	M. scrofulaceum	2	1.25%
3		Non-photochromogens 12 (7.5 %)	M.avium/ intracellulare	8	5%
			M. terrae	3	1.875%
			M. malmoense	1	0.625%
4	Rapid growers 42 (26.25%)		M. abscessus	18	11.25%
			M. fortuitum	20	12.5%
			M. chelonae	4	2.5%
5	*M. tuberculosis *78 (48.75%)			78	48.75%
	TOTAL			160	100%

**Figure 3:**
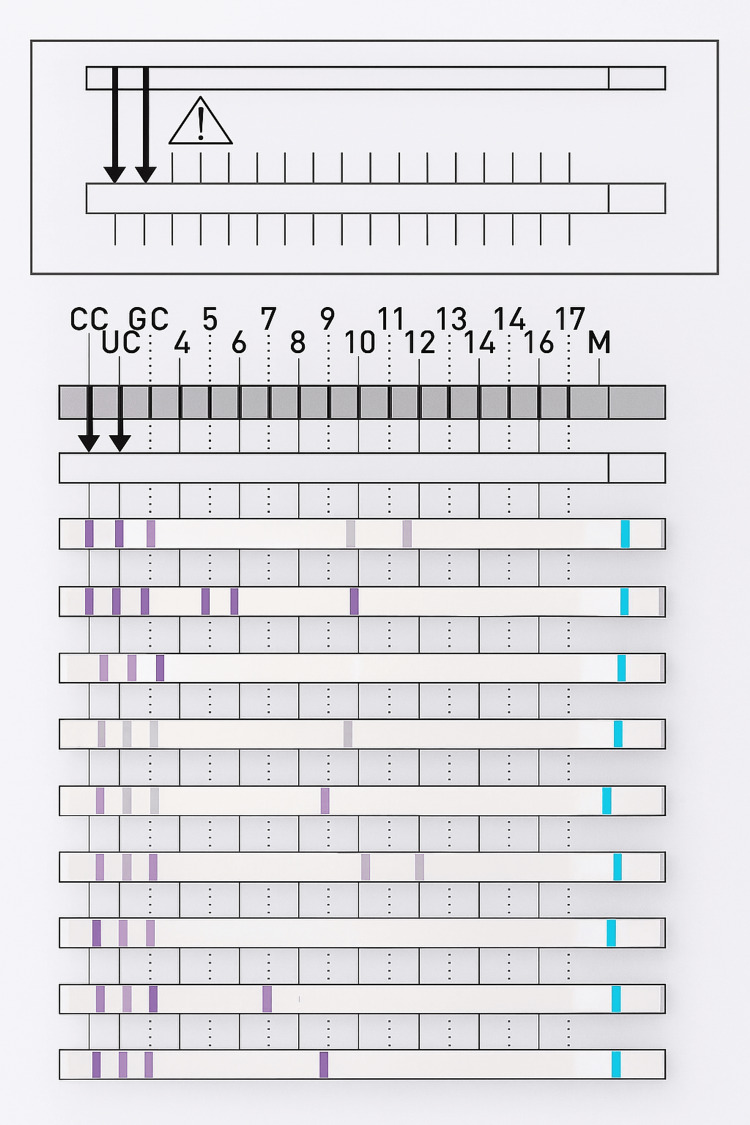
GenoType Mycobacterium CM evaluation and result sheet Bands: 1,2,3,10,12 - *Mycobacterium kansasii* 1,2,3,5,6,10 - *Mycobacterium abscessus* 1,2,3 - *Mycobacterium *species 1,2,3,9 - *Mycobacterium intracellulare* 1,2,3,7,14 - *Mycobacterium fortuitum*

**Figure 4:**
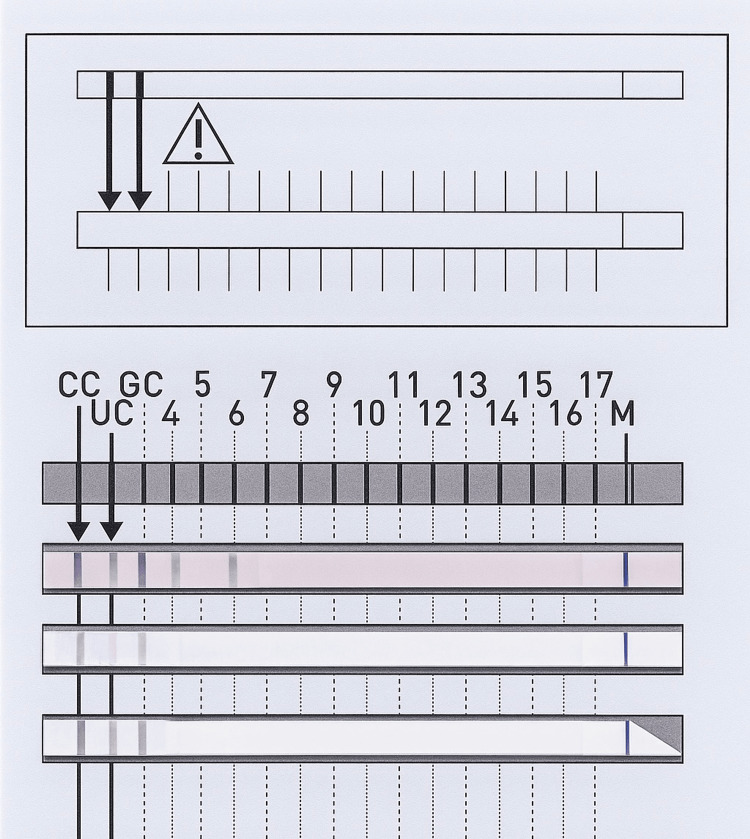
GenoType Mycobacterium AS evaluation and result sheet Bands: 1,2,3,4,6 - *Mycobacterium simiae* 1,2,3 - *Mycobacterium *species

GenoType Mycobacterium CM/AS evaluation and result sheet are shown below (Figures 3, 4)

GenoType Mycobacterium CM/AS assay results are shown in Tables 4, 5. Thirteen species which were not identified by the CM assay were subjected to the AS assay (Table 5).

**Table 4:** Species identified by GenoType Mycobacterium CM assay

S. No.	Species identified	Number	Percentage
1	M. tuberculosis complex	78	48.75%
2	M. kansasii	16	10%
3	M. scrofulaceum	2	1.25%
4	M. intracellulare	8	5%
5	M. malmoense	1	0.625%
6	M. abscessus	18	11.25%
7	M. fortuitum	20	12.5%
8	M. chelonae	4	2.5%
9	M. species	12	7.5%
10	Non Interpretable	1	0.625%
	Total	160	100%

**Table 5:** Species identified by GenoType Mycobacterium AS assay

S. No.	Species identified	Number	Percentage
1	M. simiae	10	76.92%
2	M. species	2	15.38%
3	Non interpretable (NI)	1	7.69%
	Total	13	100%

The overall concordance rate for species identification of *Mycobacterium* was 98.13% (Table 6).

**Table 6:** Concordance between the GenoType Mycobacterium CM/AS assay and phenotypic/biochemical methods for mycobacterial species identification

S. No.	Organisms	Phenotypic/ Biochemical reactions	CM/AS assay correctly identified	CM/AS assay incorrectly identified	Concordance rate
1	M. tuberculosis	78	78	0	100%
2	M. fortuitum	20	20	0	100%
3	M. abscessus	18	18	0	100%
4	M. kansasii	16	16	0	100%
5	M. simiae	10	10	0	100%
6	M. avium/ M. intracellulare	8	8	0	100%
7	M. chelonae	4	4	0	100%
8	M. terrae	3	0	3	0%
9	M. scrofulaceum	2	2	0	100%
10	M. malmoense	1	1	0	100%
	Total	160	157	3	98.13%

The average cost and turnaround time (TAT) of the conventional phenotypic/biochemical reactions and the GenoType Mycobacterium CM/AS assay are summarized in Table 7.

**Table 7:** Comparison of cost and turnaround time (TAT) between conventional phenotypic/biochemical reactions and the GenoType Mycobacterium CM/AS assay.

Method	Average cost	Average TAT	Remarks
Phenotypic/biochemical methods	Rs 1000	4-6 weeks	Labour-intensive, longer duration
GenoType CM/AS assay	Rs 2500	2-3 days	Rapid

The sensitivity, specificity, PPV and NPV of the GenoType Mycobacterium CM/AS assay were observed as 100% for all the species except *M. terrae, *for which both sensitivity and PPV was 0% while specificity and NPV was 100% and 98.13% respectively. 

For the *Mycobacterium avium *complex (MAC), the biochemical tests identified isolates as members of the complex but could not differentiate between *M. avium* and *M. intracellulare*. The 100% correlation we report with the GenoType CM/AS assay therefore refers to identification at the complex level. The GenoType CM/AS assay subsequently allowed further species-level differentiation within the complex, such as *M. intracellulare*, which is beyond the capacity of conventional biochemical testing.

Hence the overall sensitivity of the GenoType Mycobacterium CM/AS assay was 98.13% and the specificity was 100%.

## Discussion

The present study was conducted on culture isolates, and we evaluated the performance of GenoType Mycobacterium CM/AS assay in comparison with conventional phenotypic and biochemical reactions for species identification of *Mycobacterium*. In our study, isolates were grown on both solid and liquid culture media. The GenoType Mycobacterium CM/AS assay could be performed from both solid as well as liquid culture media. Most of the biochemical reactions could be performed only from the solid culture media as they require heavy and pure growth. Liquid cultures, while more sensitive and capable of yielding growth in a shorter time (seven to 14 days compared to three to eight weeks on solid media) [10,13], are more prone to contamination. Therefore, all isolates initially obtained in liquid culture were subcultured onto solid media before biochemical testing. This additional step extended the turnaround time for biochemical identification to six to seven weeks after first isolation, in contrast to the GenoType CM/AS assay, which could provide species identification within four to five days, making it more clinically useful.

Although the GenoType CM/AS assay incurs a higher cost, the benefits of rapid, reliable identification may outweigh the increased expense. Similar to our studies, several reports [8,9] have highlighted that the GenoType Mycobacterium CM/AS is highly cost-effective in high-burden settings, particularly when balanced against the economic and clinical consequences of delayed diagnosis. Thus, while the initial assay cost is higher, the reduction in diagnostic time and the potential to optimize therapy make the GenoType CM/AS a valuable diagnostic tool in routine mycobacteriology laboratories.

A total of 159 (98.75%) Mycobacterial isolates gave valid interpretable results. One isolate (*M. terrae*; 0.625%) showed an uninterpretable hybridization pattern. Such a problem was faced by other various authors like Padilla et al. [14] from Spain, who reported that 7.27% of the isolates (three MAC, three *M. terrae*, one *M. gastri* and one *M. szulgai*) gave an uninterpretable hybridization pattern and Makinen et al. (2002) from Finland [15] reported 4.93% of the isolates (one *M. avium* and three *M. intracellulare*) gave an uninterpretable hybridization pattern. Both authors have mentioned that the test requires stringent hybridization conditions to ensure that the test is carried out properly and an uninterpretable hybridization pattern may be due to inadequate hybridization temperature leading to the appearance of several nonspecific bands on the strip. When processing a large number of samples (>20), temperature does not remain optimal during the manual pipetting steps despite pre-warming of reagent and if the hybridization temperature is too low, several bands would be seen on the strips [14,15]. Large sample size and inadequate hybridization temperature could be the reason for the uninterpretable results in our study.

In our study, there were two isolates of *M. terrae* that were identified to genus level only. The GenoType assay does not include species-specific probes for *M. terrae*. Hillemann et al. from Germany in 2006 [8] and Ruiz et al. from Spain [16] were also unable to identify many isolates to the species level due to the same reason. Sometimes despite the presence of species specific probes some isolates were identified only to the genus level due to intraspecies variation occurring in the 23S rDNA target gene, and the degree of variation may depend on the geographical region and origin of the isolates as reported by Makinen et al. in 2006 from Finland [15] and Lee et al. from Australia [17].

Recently, in a multicentric study, the frequencies of different NTM species were estimated over a 20-year period in which the NTM species most frequently isolated in 1991 to 1996 were the *M. avium - M. intracellulare *complex (29.1%), *M. gordonae* (18.8%), *M. xenopi *(19%), *M. kansasii* (10.3%) and *M. fortuitum* (9.8%), accounting for approximately 87% of all NTM species [6,8]. Singh et al. [6] from India reported that the GenoType Mycobacterium CM/AS assay was able to identify 96.7% of NTM species to species level correctly. In our study too GenoType Mycobacterium CM/AS assay was able to identify 96.3% of NTM species to species level correctly.

In our study the most frequently isolated NTM species was *M. fortuitum*, which accounted for 24.39% of the NTM isolates. This was in agreement with studies from India [18].

In our study, GenoType Mycobacterium CM/AS assay showed concordance of 98.13%, with biochemical reactions taken as a gold standard. There are no studies till date where they have taken just biochemical reactions as a gold standard but few reports are available where along with biochemical reactions various other tests were taken as the gold standard such as Padilla et al. from Spain [14] used conventional biochemical methods, gas liquid chromatography, thin-layer chromatography and the AccuProbe (Bio-Merieux, Marcy-l'Étoile, France) system as a gold standard and showed concordance of 98% which is the highest concordance reported till date. Ruiz et al. from Spain [16] used conventional biochemical tests, DNA probes, and HPLC as the gold standard and concordance was found to be 94.9%. There is one study from Germany by Hillemann et al. who used sequencing of the 5' region of the 16S rRNA gene and biochemical tests as a gold standard and reported concordance for CM and AS separately, CM 92.6% and AS 89.9% [8].

Makinen et al. in 2006 from Finland [19] reported concordance of 96% which is similar to our studies but they used 16S rDNA sequencing as the gold standard. Few authors have reported concordance ranging from 88% - 95%, like Zhu Yumei et al. [20], Safianowska A et al. in 2010 from Poland [21] and Makinen et al. in 2002 from Finland [15] reported concordance of 90.8%, 93% and 95.1% respectively. Zhu Yumei et al. from China [20] used PCR sequencing (16S rRNA and ITS) as the gold standard. Safianowska A et al. [21] used HPLC whereas Makinen et al. in 2002 from Finland [15] used AccuProbe and 16S rDNA sequencing as the gold standard.

Regarding the sensitivity and specificity of the GenoType Mycobacterium CM/AS assay for species identification of mycobacteria, very few reports are available. George S et al. [22] reported that the GenoType Mycobacterium CM/AS assay had 98.23% sensitivity, 50% specificity, 99.56% PPV and 20% NPV when compared to HPLC considering biochemical test as the gold reference standard.

The lowest sensitivity of 92.7% has been reported from Spain by Padilla et al. [14]. Russo et al. from Italy [23] reported CM and AS sensitivity and specificity separately, 92.4% and 97.9% for CM, while AS sensitivity and specificity were found to be bit more, 99.3% and 99.4%. In our study, sensitivity, specificity, PPV and NPV were found to be 100% for all the mycobacterial isolates except *M. terrae*, where sensitivity and NPV were 0% but specificity was 100% and PPV was 98.13%. This may be due to the fact that the GenoType Mycobacterium CM/AS kit lacks the species-specific probe for *M. terrae*. So overall sensitivity, specificity of GenoType Mycobacterium CM/AS assay in our study was 98.13% and 100% respectively. Differences in reference “gold standards” used in various studies could contribute to reported differences in the diagnostic performance of the assay [17].

Limitation of this assay

The present study has certain limitations. The infrastructure required for line probe assays (LPA) is not available in all microbiology laboratories but as the National Tuberculosis Elimination Program (NTEP) reference laboratory has a facility for LPA, these tests can be done in the NTEP laboratory. Though the test requires technical expertise and the person should be trained in molecular biology methods, but LPA is already ongoing in NTEP labs so can be performed there. A further limitation is that the GenoType CM/AS assay is restricted to probes for the most common and clinically relevant NTM species, and hence cannot identify all NTM [6]. In our study also, due to the absence of a probe for *M. terrae*, its identification to species level was missed but this could be easily overcome by the inclusion of the species-specific probe for these species too by the manufacturer. A further limitation is the relatively higher cost of the assay compared to conventional methods, which may restrict its routine application in resource-limited settings despite its diagnostic advantages. Finally, we did not perform sequencing, which remains the gold standard for species identification and would have provided a more definitive comparison with the results of the LPA. 

## Conclusions

GenoType Mycobacterium CM/AS assay has an excellent concordance rate, sensitivity and specificity for species identification of mycobacteria. The test is able to identify most of the clinically relevant, common *Mycobacterium* species and some additional less common species with a short turnaround time. It is simple, rapid, easy to interpret and can easily be implemented in routine work for mycobacterial species differentiation in the clinical setting of NTEP laboratories where infrastructure for LPA exists. Unlike conventional biochemical tests, which are labor-intensive, time-consuming, and not routinely performed in many laboratories, the GenoType CM/AS assay makes use of existing LPA infrastructure, making it practical for widespread use. Importantly, in resource-limited settings where advanced molecular methods such as sequencing or matrix-assisted laser desorption/ionization - time of flight (MALDI-TOF) are not readily available, this assay provides a dependable alternative for reliable species-level identification. It will directly help the patients in targeted therapy and management of infections caused by different mycobacterial species and indirectly it will help in reducing development of antimicrobial drug resistance in the community.
